# NetDiff – Bayesian model selection for differential gene regulatory network inference

**DOI:** 10.1038/srep39224

**Published:** 2016-12-16

**Authors:** Thomas Thorne

**Affiliations:** 1 Division of Brain Sciences, Imperial College London, UK

## Abstract

Differential networks allow us to better understand the changes in cellular processes that are exhibited in conditions of interest, identifying variations in gene regulation or protein interaction between, for example, cases and controls, or in response to external stimuli. Here we present a novel methodology for the inference of differential gene regulatory networks from gene expression microarray data. Specifically we apply a Bayesian model selection approach to compare models of conserved and varying network structure, and use Gaussian graphical models to represent the network structures. We apply a variational inference approach to the learning of Gaussian graphical models of gene regulatory networks, that enables us to perform Bayesian model selection that is significantly more computationally efficient than Markov Chain Monte Carlo approaches. Our method is demonstrated to be more robust than independent analysis of data from multiple conditions when applied to synthetic network data, generating fewer false positive predictions of differential edges. We demonstrate the utility of our approach on real world gene expression microarray data by applying it to existing data from amyotrophic lateral sclerosis cases with and without mutations in C9orf72, and controls, where we are able to identify differential network interactions for further investigation.

There has been much work in the literature on the inference of networks from gene expression data, utilising a variety of approaches including tests for correlation^1^, graphical models^2^,^3^, and mutual information based approaches^4^. Graphical modelling approaches have the advantage that they consider conditional independence, so that regulatory network interactions are only predicted where the other explanatory variables are unable to account for all of the variation observed.

A task that has received less attention, but would allow for greater exploitation of the experimental data that is often generated, is the inference of differential networks^5^,^6^,^7^,^8^,^9^. The aim of differential network inference approaches is similar to that of tests for differential expression, but rather than detecting changes in the expression of a single gene, the aim is to infer changes in the regulatory network structure itself between multiple conditions. This allows for more specific mechanistic insights into the molecular processes underlying changes between, for example, cases and controls in a disease study^6^,^8^.

In previous work we have explored the application of flexible non-parametric models of regulatory networks that allow for changes in network structure with an arbitrary number of networks^8^. However this flexibility adds complexity to the model that does not necessarily deliver greater insights, at the expense of a complex and computationally demanding implementation. In a Bayesian framework, a parsimonious approach would seem to be applying Bayesian model selection to compare a model allowing for a change in the network structure between conditions against a model of a static network. Bayesian model selection approaches automatically compensate for changes in the number of parameters between models, so that a more complex model will not always improve on a simpler nested model by overfitting the data.

Although differential networks can be learnt through the independent analysis of samples of gene expression data from multiple conditions, as the process suffers from the “*large p small n*” problem, it would be expected that a substantial proportion of the differential edges observed would be a side-effect of false positive or false negative predictions. Instead we propose to compare a model of no change between conditions, against a model where edges are allowed to vary, for each gene independently. This allows the network edges of genes whose interactions do not vary to be learnt more robustly by utilising the data from both conditions, whilst where there is evidence of a change in the network, differential edges are identified.

Here we propose a fast and easily applicable method to perform Bayesian model selection on gene expression microarray data collected between multiple conditions, and demonstrate an application of this method to publicly available gene expression microarray data from Amyotrophic Lateral Sclerosis (ALS) cases and controls, investigating the impact of C9orf72 GGGGCC repeat expansions in the underlying regulatory network by comparing patients with and without these mutations.

## Methods

### Gaussian graphical models

In a Gaussian Graphical Model (GGM) a set of random variables *X*^1^, …, *X*^*p*^ are assumed to take a multivariate normal distribution so that 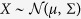. Then in a graphical representation of such a model, pairs of nodes *i* and *j* have no edge between them if and only if the two corresponding random variables are conditionally independent, or equivalently 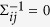. Identifying such relationships is possible using the partial correlation between pairs of random variables *X*^*i*^ and *X*^*j*^ with respect to the remaining variables *Z*^−*i*,*j*^, defined as


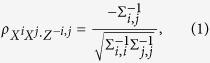


so that an edge from variable *i* to *j* exists in the graphical model iff 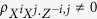. Unfortunately in many cases of interest, such as the analysis of gene expression microarray data, we are faced with the so called “*large p small n*” problem, characterised by a large number of variables *p* and a comparatively small number of observations *n*. In such cases it is not possible to directly calculate the partial correlations between the variables as the empirical covariance matrix will be singular. Fortunately however there are a number of ways that we can determine the partial correlations in such cases^10^.

One such method to infer the structure of a GGM when we are faced with more variables than observations is to use regularised regression methods to infer the partial correlations between the nodes. If the vector of *n* observations of variable *i* is denoted *Y* = *X*^*i*^ and the remaining variables as *X*^−*i*^ = *X*^*j*≠*i*^, then we seek to infer the posterior of *β*^*i*^ with


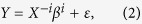


where 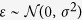. Then given *β*^*i*^ for each variable *i*, we can estimate the partial correlations between variables *i* and *j* as ref. 10


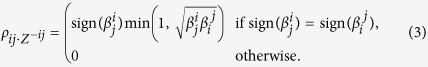


Here we apply the regularised regression method introduced in Caron *et al*.^11^, which has been demonstrated to outperform the lasso and other regularised regression methods. Sparsity is enforced by introducing a scale mixture of Gaussian distributions as the prior on the regression coefficients *β*_*w*_^11^ (omitting indices of *β, ζ*^2^ and *σ*^2^ over the *m* variables for clarity),


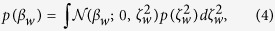


with each 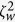 in turn distributed as an inverse Gaussian,


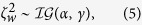


where the parameters *α* and *γ* control the penalty placed on the *β*_*w*_, here set to 0.1 and 1 respectively. We place a vague gamma prior on *σ*^2^. To evaluate equation 3 we take the maximum a posteriori (MAP) estimates of 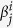 to derive partial correlations *ρ*. Significant partial correlations are identified using the fdrtool R package^12^ to estimate the local false discovery rate, with a cutoff of 0.025.

### Differential networks

To test for variation in the network structure between conditions, we allow the *n* observations to be partitioned into *K* sets, *X*_1_, …, *X*_*K*_. Here we focus on the case where *K* = 1 or *K* = 2, being models of a single network for all observations, and a network varying between two conditions, respectively. Considering the regression approach described in the previous section it is straightforward to allow for independent network structures between the *K* sets of observations,


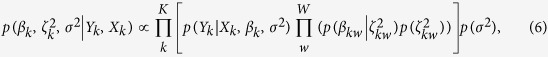


and for a given model *M* we can then consider the model evidence,


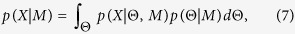


where the model parameters are subsumed into Θ. Comparing the two competing models *K* = 1 and *K* = 2, we can calculate a Bayes factor 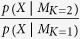 to assess if there is significant evidence for preferring one model over the other, and in our method we take a Bayes factor larger than 2 as support for model *M*_*K*=2_. Having calculated Bayes factors for each variable *X*_1_, …, *X*_*p*_ we can derive partial correlations for the two conditions *k* = 1 and *k* = 2 from the appropriate *β*_*kw*_.


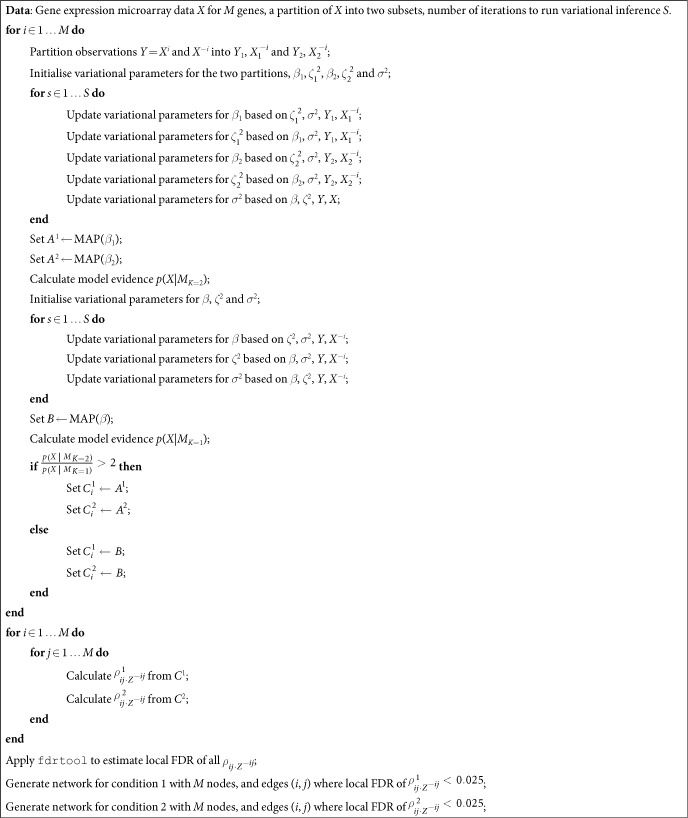


**Algorithm 1:** Outline of the inference procedure. Variational parameter updates and model evidence are given in the Supplementary methods. MAP refers to the Maximum A Posteriori value.

### Implementation

To allow us to derive approximate posterior distributions of the model parameters, and perform model selection by estimating the model evidence, we apply a variational Bayes^13^,^14^,^15^,^16^,^17^ inference scheme to our model. An outline of the methodology is given in Algorithm 1, and derivations of the variational updates given in the Supplementary methods.

A benefit of the variational inference approach is that as well as providing a fast approximation of the posterior distribution, it allows for the evaluation of a lower bound on the log evidence *p(x*|*M*) for a model *M*, used as an approximation to *p(x*|*M*) in calculating the Bayes factor. The model evidence *p(x*|*M*) is not typically available when utilising Markov Chain Monte Carlo (MCMC) methods, and so model selection using MCMC methods typically requires more complex approaches such as Reversible Jump MCMC^18^.

The method described was implemented as a package for the R Statistical Computing Environment^19^ and is available from https://gitlab.com/tt104/NetDiff. In benchmarking the method we also apply the GeneNet^2^ and FastGGM^3^ R packages.

### Synthetic network benchmarking

**Figure 1:**
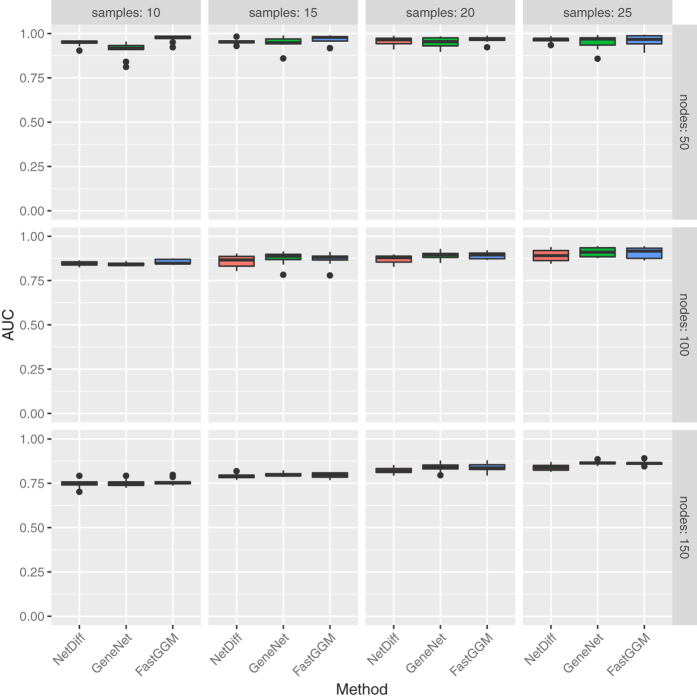
Boxplots of Area Under Curve (AUC) of the receiver operating characteristic curve for each of the methods compared on random networks, with various numbers of network nodes and simulated sample data points from each network.

**Figure 2:**
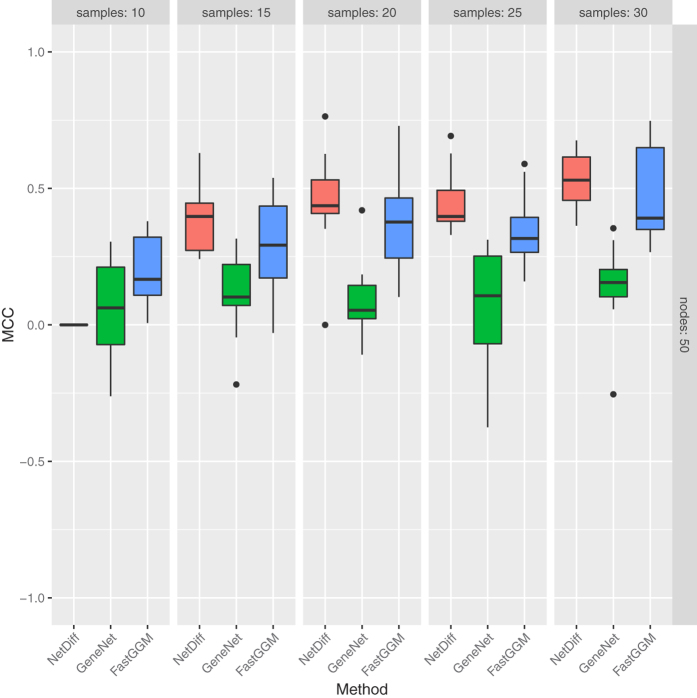
Boxplots of Matthews Correlation Coefficient (MCC) for each of the methods applied to random networks with structures varying between conditions, with various different numbers of samples per condition.

**Figure 3:**
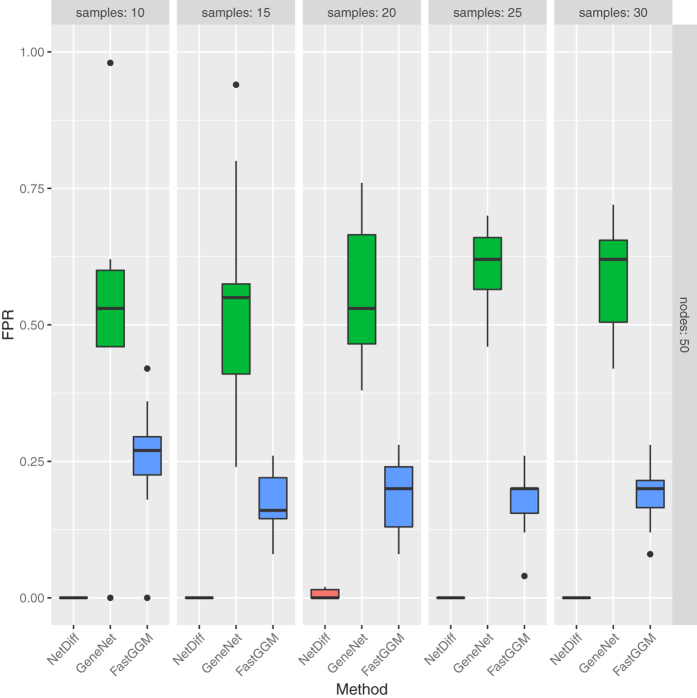
Boxplots of False Positive Rate (FPR) for each of the methods applied to random networks with structures remaining identical between conditions, with various different numbers of samples per condition.

The GeneNet^2^ R package was used to simulate partial correlations between sets of variables of varying sizes, and to generate observations of the variables under these network structures. To assess performance in network inference we calculate the area under the Receiver Operating Characteristic (ROC) curve using the PRROC R package^20^. This was repeated for multiple synthetic network structures to generate the distributions of metrics shown in the boxplots in Figs 1, 2 and 3.

## Results

### Synthetic data

We compare our approach to two existing methods from the literature to learn Gaussian graphical models from gene expression microarray data, GeneNet and FastGGM. The running time of our method, and of GeneNet and FastGGM was benchmarked on the synthetic data, see Supplementary Fig. 1. Although our method is slower, the running times for all methods are short, on the order of seconds, so that in practice this will be unlikely to have any real impact. The variational inference approach is considerably more computationally efficient when compared to MCMC approaches, for example in a previous method we developed applying a similar sparse regression method in a Gibbs sampling framework, inference took around 20 hours running in parallel on a 12 core CPU^8^.

#### Network inference

Applying our method to synthetic data with varying network sizes and numbers of observations, we see from the plots in Fig. 1 that our method performs well in terms of the area under the ROC curve, and is comparable to the other approaches that were benchmarked. Although the performance of our method does not exceed that of competing methods, by working in a Bayesian framework we are easily able to expand the approach to perform Bayesian model selection, as described in the following section. There are further benefits to the Bayesian approach, such as the straightforward inclusion of prior information. As expected, the performance of the various approaches decreases as the number of samples is reduced, and also falls as the size of the network grows for a given number of samples.

#### Differential networks

We test the main focus of our method, the learning of differential networks though Bayesian model selection, by generating synthetic data sets where the network structure is known to change for a subset of nodes between two sets of samples, in networks of 50 nodes. To benchmark our method we calculate the Matthews Correlation Coefficient (MCC), comparing the nodes with predicted changes to the known set of positives. Matthews correlation coefficient allows us to test the performance of our method at a specific cut-off, without concerns about effects of class imbalances. To test our hypothesis that a method performing integrated analysis between conditions and attempting to identify evidence for a change in network structure will perform better than independent analyses of each condition, we also compare the performance of GeneNet and FastGGM in identifying nodes whose network interaction partners change between conditions, taking significant edges as those with a local false discovery rate under 0.025 and testing for changes in the inferred networks between conditions.

From the results shown in Fig. 2, it is apparent that for 15 or more samples our approach outperforms the alternative methods, demonstrating higher Matthews correlation coefficient values than GeneNet, and to a lesser extent FastGGM. Our approach has less variance in performance than FastGGM, likely due to the greater susceptibility to noise inherent in learning differential networks from independent analyses. However our method does require a relatively large number of samples per condition to effectively predict changes in the network structure, as can be seen in the case for 10 samples.

To further test the robustness of our approach, we also apply the method to synthetic data from two conditions, where the network structure does not change between conditions, as shown in Fig. 3. As there are no true positive differential edges, we only measure the false positive rate, which for our method is significantly lower than applying GeneNet or FastGGM to each condition independently. This is to be expected, as our method is able to identify that there is no change in the network structure, and learn using data from both conditions combined, whilst the other approaches must learn networks independently from each condition, and differential edges may be erroneously identified simply due to noise in the output.

### Dysregulation of gene expression in ALS patients with C9orf72 mutations

To test the applicability of our approach to real world datasets we applied our method to the data of Cooper-Knock *et al*.^21^, Gene Omnibus accession *G*SE68607, consisting of gene expression microarray data collected from lymphoblastoid cell lines derived from 54 ALS patients and 15 controls. The ALS patients are further divided into 31 with C9orf72 mutations and 23 without. Hexanucleotide repeat expansions in C9orf72 have been shown to cause familial ALS^22^, but the exact mechanisms are not understood. To identify the causal mechanisms by which these expansions lead to ALS, we seek to investigate the differences in gene regulation between controls and ALS cases with and without expansions. We do so by comparing gene expression microarray data from each subclass of ALS patients to one another, and to controls, identifying differential network edges using our proposed method. To render the analysis tractable we choose a subset of the genes in the dataset to focus on in our study, specifically those in the KEGG database^23^,^24^ ALS pathway, accession *h*sa05014, as well as C9orf72.

**Figure 4:**
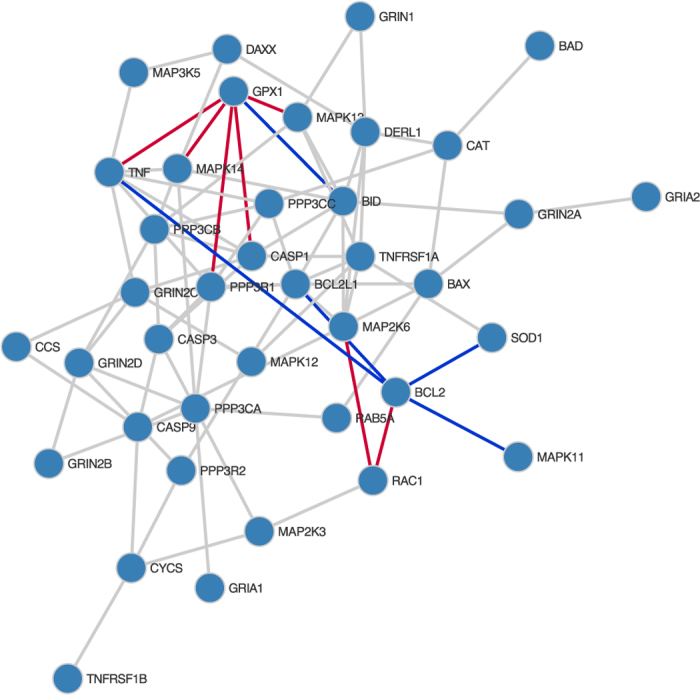
Differential network comparing ALS cases without C9orf72 mutations and controls. Conserved network edges are shown in grey, whilst edges only present in ALS cases are in red, and those only present in controls are in blue.

Firstly considering the differential networks inferred between cases without C9orf72 mutations and controls, as shown in Fig. 4, we see that whilst the majority of network edges are conserved, there appear to be a small number of genes whose interactions change considerably. Several regulatory interactions are gained by GPX1, to PPP3R1, CASP1, TNF, all promoters of apoptosis, and MAPK13 and MAPK14. The interaction between BID and GPX1 is only observed in controls. BCL2 loses interactions with SOD1, BCL2L1, TNF and MAPK11, whilst gaining an interaction with RAC1. Interestingly, BCL2 is crucial in inhibiting apoptosis, and has been demonstrated to have reduced expression in ALS in mice^25^. RAC1, thought to be a key player in immune response in ALS^26^, also gains an interaction with MAP2K6.

**Figure 5:**
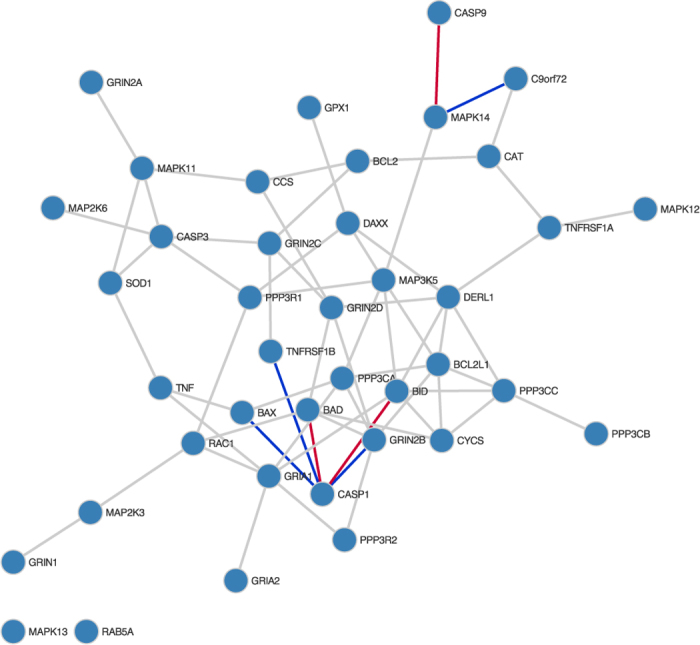
Differential network comparing ALS cases with and without C9orf72 mutations. Conserved network edges are shown in grey, whilst edges only present in ALS cases with C9orf72 mutations are in blue, and those only present in ALS cases without C9orf72 mutations are in red.

Changes within the set of ALS patients, between those with and without mutations in C9orf72, are less pronounced, as seen in Fig. 5. Interestingly cases with a C9orf72 mutation gain an interaction with MAPK14, whilst MAPK14 and CASP9 do not interact in cases with a C9orf72 mutation. Furthermore CASP1 gains interactions with BAX, TNFRSF1B and GRIN2B, whilst losing interactions with BAD and BID. CASP1, BAD, BAX and BID all promote apoptosis, whilst TNFRSF1B inhibits apoptosis. GRIN2B encodes NR2B, which activates apoptosis when overexpressed^27^.

**Figure 6:**
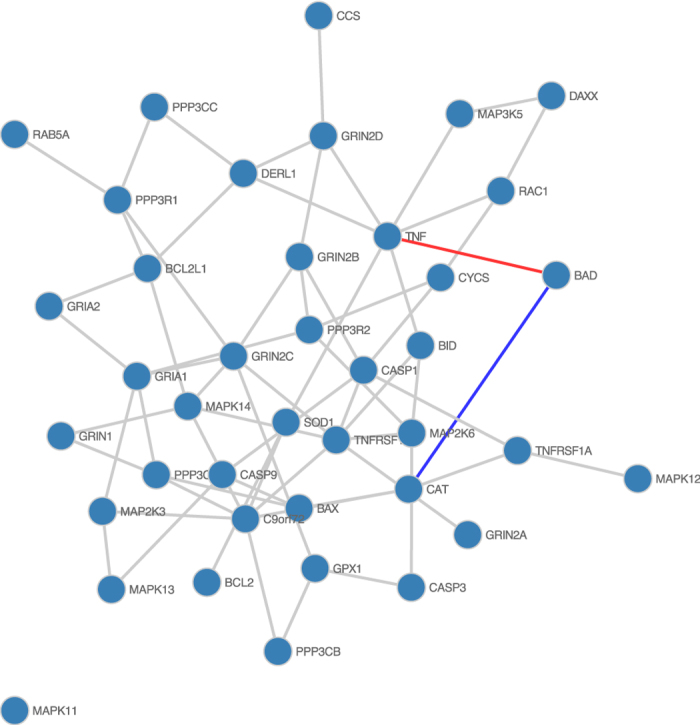
Differential network comparing ALS cases with C9orf72 mutations and controls. Conserved network edges are shown in grey, whilst edges only present in ALS cases with C9orf72 mutations are in red, and those only present in controls are in blue.

We also compare patients with mutations in C9orf72 with controls, again giving less pronounced results, shown in Fig. 6. In patients with C9orf72 mutations, BAD appears to lose an interaction with CAT, and gain one with TNF. As mentioned previously TNF is a promoter of apoptosis, again showing dysregulation of apoptosis as a potential mechanism of ALS^28^.

Genes having gene ontology annotations for positive regulation of apoptosis were found to be significantly differentially expressed in microarray data from lymphoblastoid cell lines derived from ALS cases with C9orf72 mutations^21^, and our results suggest that there may be differences in regulation of apoptosis between ALS cases with and without C9orf72 mutations.

It should be highlighted that the network edges (interactions) in inferred graphical models from gene expression data do not necessarily correspond to direct regulatory interactions in the form of transcription factor binding, but may take the form of the indirect modulation of transcription through the action of intermediary steps that are not measured by transcriptomic studies.

## Discussion

We demonstrate that our Bayesian model selection approach is able to identify changes in network structures between conditions. Whilst the performance of our approach in learning the network structure under a single condition does not improve on competing methods, the focus is in this work to allow for the inference of differential networks to allow gene expression microarray data from case control studies to be fully exploited. The model selection approach we describe here is enabled by the variational inference scheme we employ that allows us to estimate the model evidence in a computationally efficient manner, and we show that it allows us to more robustly identify differential regulatory interactions between conditions. Due to the challenging nature of the task of learning a network from microarray data, likely introducing noise in the results, an integrated approach that can utilise all of the samples whilst testing for changes in the network structure is preferable to independent learning on cases and controls, as we demonstrate here.

## Additional Information

**How to cite this article**: Thorne, T. NetDiff – Bayesian model selection for differential gene regulatory network inference. *Sci. Rep.* **6**, 39224; doi: 10.1038/srep39224 (2016).

**Publisher's note:** Springer Nature remains neutral with regard to jurisdictional claims in published maps and institutional affiliations.

## Supplementary Material

Supplementary Materials
