# Supplementary material for: NetDiff – Bayesian model selection for differential gene regulatory network inference
Source: Sci Rep. 2016 Dec 16;6:39224. doi: 10.1038/srep39224 (PMC5159802; doi:10.1038/srep39224)
Supplement: Supplementary Materials [file srep39224-s1.pdf]

Supplementary materials: NetDiff – Bayesian model selection for differential gene regulatory network inference.

Thomas Thorne

Supplementary methods

Mean field approximation

For a single variable the posterior distribution is approximated as a mean field approximation on the parameters

$$\prod_k^K \left[p(Y_k|X_k, \beta_k, \sigma^2) \prod_w^W \left(p(\beta_{kw}|\zeta_{kw}^2) p(\zeta_{kw}^2) \right) \right] p(\sigma^2) \approxeq \prod_k^K q(\beta_k) \prod_w^W q(\zeta_{kw}) q(\sigma^2) \quad (1)$$

The optimal distributions $\hat{q}$ can be derived, firstly for β_k, the regression coefficients:

$$\begin{aligned} \log \hat{q}(\beta_k) &= \mathbb{E}_{q_{-\beta}} \left[\log p(Y_k|X_k, \beta_k, \sigma^2) \prod_w^W p(\beta_{kw}|\zeta_{kw}^2) \right] + \text{const.} \\ &= \mathbb{E}_{q_{-\beta}} \left[-\frac{1}{2\sigma^2} (Y_k - X_k\beta_k)^T (Y_k - X_k\beta_k) - \frac{1}{2} \beta_k^T \Omega \beta_k \right] + \text{const.} \\ &= -\frac{1}{2} \mathbb{E}_{q_{-\beta}} \left[\frac{1}{\sigma^2} \right] \beta_k^T X_k^T X_k \beta_k - \frac{1}{2} \beta_k^T \Omega_k \beta_k + \mathbb{E}_{q_{-\beta}} \left[\frac{1}{\sigma^2} \right] \beta_k^T X_k^T Y_k + \text{const.} \end{aligned} \quad (2)$$

$$\hat{q}(\beta_k) \sim \mathcal{N}(\mu_k, \Sigma_k) \tag{3}$$

$$\mu_k = \Sigma_k \mathbb{E}_q \left[\frac{1}{\sigma^2}\right] X_k^T Y_k \tag{4}$$

$$\Sigma_k = \left(\mathbb{E}_q \left[\frac{1}{\sigma^2}\right] X_k^T X_k + \Omega_k\right)^{-1} \tag{5}$$

$$\Omega_k = \mathrm{diag}\left(\mathbb{E}_q \left[\frac{1}{\zeta_{kw}^2}\right]\right) \tag{6}$$

$$\mathbb{E}_q[\beta_{kw}^2] = \mu_{kw}^2 + \mathrm{diag}(\Sigma_k)_w. \tag{7}$$

Then considering the scaling parameters ζ_{kw} for each regression coefficient β_{kw}, omitting the index k for clarity:

$$\begin{aligned}
\log \hat{q}(\zeta_w^2) &= \mathbb{E}_{q_{-\zeta_w}} \left[\log p(\beta_w|\zeta_w^2) p(\zeta_w^2)\right] + \text{const.} \\
&= \mathbb{E}_{q_{-\zeta_w}} \left[-\frac{1}{2}\log \zeta_w^2 - \frac{\beta_w^2}{2\zeta_w^2} - \frac{3}{2}\log(\zeta_w^2) - \frac{1}{2}(\gamma^2 \zeta_w^2 + \frac{\alpha^2}{\zeta_w^2})\right] + \text{const.} \\
&= -2\log(\zeta_w^2) - \frac{1}{2}\left(\gamma^2\zeta_w^2 + \frac{\alpha^2 + \mathbb{E}_{q_{-\zeta_w}}[\beta_w^2]}{\zeta_w^2}\right) + \text{const.}
\end{aligned} \tag{8}$$

$$\hat{q}(\zeta_w^2) \sim \mathcal{GIG}(a_w, b_w, -1) \tag{9}$$

$$a_w = \gamma^2 \tag{10}$$

$$b_w = \alpha^2 + \mathbb{E}_q[\beta_w^2] \tag{11}$$

$$\mathbb{E}_q[\zeta_w^2] = \sqrt{\frac{b_w}{a_w}} \frac{\mathcal{K}_0(\sqrt{a_w b_w})}{\mathcal{K}_{-1}(\sqrt{a_w b_w})} \tag{12}$$

$$\mathbb{E}_q\left[\frac{1}{\zeta_w^2}\right] = \sqrt{\frac{a_w}{b_w}} \frac{\mathcal{K}_2(\sqrt{a_w b_w})}{\mathcal{K}_1(\sqrt{a_w b_w})} \tag{13}$$

$$\mathbb{E}_q[\log \zeta_w^2] = \frac{1}{2}\log\frac{b_w}{a_w} - \frac{\mathcal{K}_0(\sqrt{a_w b_w})}{\sqrt{a_w b_w}\mathcal{K}_1(\sqrt{a_w b_w})} \tag{14}$$

where $\mathcal{K}$ is the modified Bessel function of the second kind. Finally for the variance of the error term we can derive:

$$\begin{aligned}
\log \hat{q}(\frac{1}{\sigma^2}) &= \mathbb{E}_{q_{-\sigma}} \left[\log \prod_k^K p(Y_k|X_k, \beta_k, \sigma^2) p(\sigma^2)\right] + \text{const.} \\
&= \mathbb{E}_{q_{-\sigma}} \Bigg[\sum_k^K \left(\frac{L_k}{2}\log(\frac{1}{\sigma^2}) - \frac{1}{2\sigma^2}(Y_k - X_k\beta_k)^T (Y_k - X_k\beta_k)\right) \\
&\qquad + (s-1)\log(\frac{1}{\sigma^2}) - \frac{t}{\sigma^2}\Bigg] + \text{const.}
\end{aligned} \tag{15}$$

$$\hat{q}(\frac{1}{\sigma^2}) \sim \mathrm{Gamma}\,(u, v) \tag{16}$$

$$u = s + \frac{\sum_k^K L_k}{2} \tag{17}$$

$$v = t + \frac{\sum_k^K \left(\mathbb{E}_q[(Y_k - X_k\beta_k)^T(Y_k - X_k\beta_k)]\right)}{2} \tag{18}$$

The variational parameters are iteratively optimised to find the optimal approximate posterior distribution.

Variational lower bound

To perform model selection we utilise the variational lower bound on the model evidence, which can be calculated using

$$\begin{aligned}\log \mathcal{L}(q) = \mathbb{E}_q \left[\log \prod_k^K \left(p(Y_k|X_k, \beta_k, \sigma^2) \prod_w^W p(\beta_{kw}|\zeta_{kw}^2) p(\zeta_{kw}^2) \right) p(\sigma^2) \right] \\ - \mathbb{E}_q \left[\log \prod_k^K \left(q(\beta_k) \prod_w^W q(\zeta_{kw}) \right) q(\sigma^2) \right].\end{aligned} \tag{19}$$

The various terms of which are

$$\mathbb{E}_q[\log p(Y_k|X_k, \beta, \sigma^2)] = \mathbb{E}_q \left[\sum_k^K - \frac{L_k}{2} \log 2\pi\sigma^2 - \frac{1}{2\sigma^2}(Y_k - X_k\beta_k)^T(Y_k - X_k\beta_k) \right] \tag{20}$$

$$\mathbb{E}_q \left[\log p(\beta_{kw}|\zeta_{kw}^2)\right] = \mathbb{E}_q \left[-\frac{1}{2}\log 2\pi\zeta_{kw}^2 - \frac{1}{2\zeta_{kw}^2}\beta_{kw}^2 \right] \tag{21}$$

$$\mathbb{E}_q \left[\log p(\zeta_{kw}^2)\right] = \mathbb{E}_q \left[\log\frac{\alpha}{2\pi} + \alpha\gamma - \frac{3}{2}\log \zeta_{kw}^2 - \frac{1}{2}(\gamma^2\zeta_{kw}^2 + \frac{\alpha^2}{\zeta_{kw}^2}) \right] \tag{22}$$

$$\mathbb{E}_q[\log p(\sigma^2)] = \mathbb{E}_q \left[s\log t - \log\Gamma(s) + (s-1)\log\frac{1}{\sigma^2} - \frac{t}{\sigma^2} \right] \tag{23}$$

$$-\mathbb{E}_q[\log q(\beta_k)] = \frac{M}{2}(\log 2\pi + 1) + \frac{1}{2}\log|\Sigma_k| \tag{24}$$

$$-\mathbb{E}_q[\log q(\zeta_w^2)] = \frac{(2 - a_w b_w)\mathcal{K}_0(\sqrt{a_w b_w})}{\sqrt{a_w b_w}\mathcal{K}_1(\sqrt{a_w b_w})} - \log\left(\frac{2b_w\mathcal{K}_1(\sqrt{a_w b_w})}{\sqrt{a_w b_w}}\right) - 1 \tag{25}$$

$$-\mathbb{E}_q[\log q(\sigma^2)] = u - \log v + \log[\Gamma(u)] + (1-u)\psi(u) \tag{26}$$

where the expectations under q are as provided in the mean field approximation section and ψ is the digamma function.

Supplementary figures

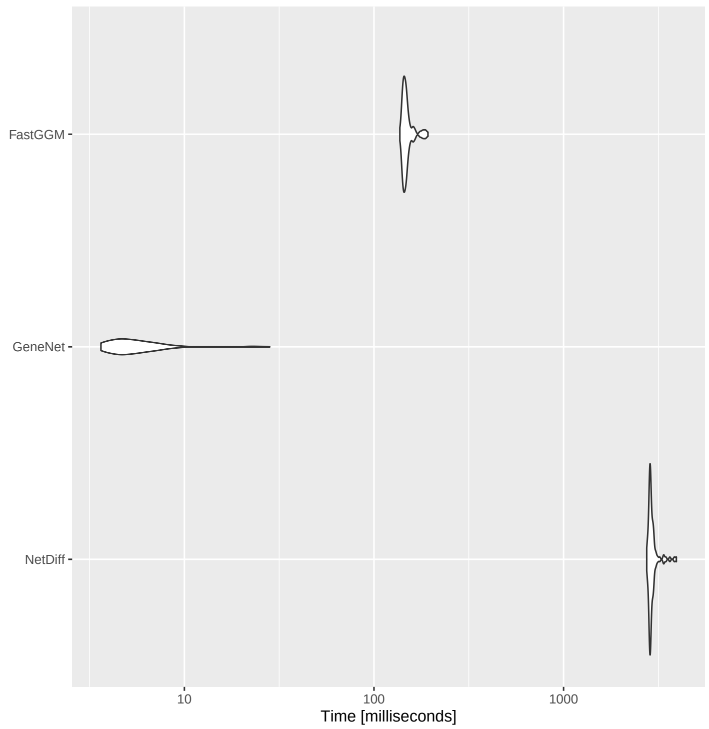

Figure 1: Distributions of timings from multiple runs of the three methods compared when run on a dataset consisting of 50 genes, measured using the `microbenchmark` R package.
